# Pathological characteristics analysis of children with intermittent and persistent hydronephrosis due to uretero-pelvic junction obstruction

**DOI:** 10.3389/fped.2024.1416789

**Published:** 2024-07-23

**Authors:** Ma Yan, Zou Jizhen, Xiao Ping, Huang Cheng, Bai Dongsheng

**Affiliations:** ^1^ Department of Urology, Children's Hospital, Capital Institute of Pediatrics, Beijing, China; ^2^ Department of Pathology, Children's Hospital, Capital Institute of Pediatrics, Beijing, China

**Keywords:** uretero-pelvic junction obstruction, intermittent hydronephrosis, pathology, Cajal-like cells, children

## Abstract

**Objective:**

To analyze from a pathological perspective the differences between intermittent and persistent hydronephrosis in children with uretero-pelvic junction obstruction.

**Methods:**

23 children who underwent unilateral dismembered pyeloplasty (Anderson-Hynes operation) for intermittent hydronephrosis from September 2017 to March 2024 were included in the observation group. They were compared with a control group consisting of 23 children with persistent hydronephrosis matched for age, gender, and affected side. All children had the narrowed segment surgically excised during the operation, while other obstructive causes (such as polyps, crossing vessels, or tumor compression) were excluded. The specimens were analyzed for muscle and collagen content using Masson's trichrome staining, and the collagen-to-muscle ratio (CMR) was calculated. The number of Cajal-like cells was quantified with c-kit immunohistochemical staining. For all slides, 10 random fields of view were selected under a 400× optical microscope to record pathological data and calculate mean values. Pathological indicators between the two groups were compared using the *T*-test and the Chi-square test, with *P* < 0.05 considered statistically significant.

**Results:**

The observation group showed a significant difference in the number of fields with low, medium, and high densities of Cajal-like cells compared to the control group [132 (57.4%) vs. 173 (75.2%); 70 (30.4%) vs. 38 (16.5%); 28(12.2%) vs. 19 (8.3%), *P* < 0.001]. The uretero-pelvic junction in children with intermittent hydronephrosis had lower collagen content, higher muscle content, and a more regular arrangement. The collagen-muscle ratio was significantly lower than that in children with persistent hydronephrosis [(1.59 ± 0.65) vs. (3.98 ± 1.19), *P* < 0.001].

**Conclusion:**

Compared with persistent hydronephrosis, the narrowed segment at the uretero-pelvic junction in children with intermittent hydronephrosis has a higher density of Cajal-like cells; lower collagen content, and higher muscle content (lower collagen-muscle ratio).

## 1 Introduction

Congenital obstruction of the ureteropelvic junction (UPJO) is the most common congenital anomaly leading to pediatric hydronephrosis, with an overall incidence of 1 in 1,500 newborns (1). A subset of these patients presents with intermittent hydronephrosis, accounting for 3.4%–11% of hydronephrosis cases (2–4). Children with intermittent hydronephrosis often exhibit acute abdominal pain accompanied by nausea and vomiting as the principal clinical symptoms. During acute episodes, imaging studies such as renal ultrasonography and intravenous pyelography can demonstrate significant pelvicalyceal dilatation, while no clinical symptoms or radiological abnormalities are observed during the asymptomatic intervals (5, 6). The etiology of intermittent hydronephrosis includes compression by crossing vessels and obstruction by polyps (7, 8), and some children have strictures and poor peristalsis at the ureteropelvic junction (9–11). This study pathologically analyzed the differences in the narrowed segment of the ureteropelvic junction between children with intermittent hydronephrosis and those with persistent hydronephrosis, as reported below.

## 2 Materials and methods

### 2.1 Study subjects

Study Subjects: From September 2017 to March 2024, 23 pediatric patients who underwent unilateral dismembered pyeloplasty for intermittent hydronephrosis at our hospital were included in the observation group. They were matched with another 23 patients with persistent hydronephrosis by age, gender, and affected side to form the control group. All children were excluded from the study if they had renal developmental disorders, malrotation, or horseshoe kidney deformities. The children in the observation group exhibited intermittent hydronephrosis preoperatively, characterized by an increase in the anteroposterior diameter of the renal pelvis and thinning of the cortex during episodes, with or without Dietl's crisis. During intermittent stage, the anteroposterior diameter of the renal pelvis was almost identical to that of normal children, and all underwent dismembered pyeloplasty during episodes. Indications for surgical intervention include impaired split renal function (<40%), a decrease in split renal function of >10% in subsequent studies, poor drainage function after the administration of furosemide, increased anteroposterior diameter on ultrasound (US), and grade III and IV dilatation as defined by the Society for Fetal Urology.

### 2.2 Intraoperative findings

Both groups of children underwent dismembered pyeloplasty, including laparoscopic and open approaches. Intraoperatively, a significant stenosis at the ureteropelvic junction was found, but there was no excessively high location of the ureteropelvic junction, and compression by crossing vessels or tumors was ruled out. No polyps or valvular abnormalities were found within the lumen when the ureteropelvic junction was split.

### 2.3 Pathology techniques

The most constricted segment of the excised specimen was sectioned for Masson's staining and c-kit immunohistochemistry. Tissue samples fixed in formalin and embedded in paraffin were stained with Masson's stain, where the extracellular matrix or collagen was prominently displayed in blue, and smooth muscle in red. The ratio of blue to red areas was defined as the CM ratio.

Immunohistochemistry steps: Sections of 10% formalin-fixed paraffin-embedded tissues were subjected to high-pressure antigen retrieval using EDTA pH 9.0. Paraffin sections were deparaffinized to water, incubated with 3% hydrogen peroxide at room temperature for 10 min to quench endogenous peroxidase activity, rinsed with distilled water, soaked in PBS for 5 min, blocked with normal goat serum working solution, and incubated at room temperature for 10 min. After discarding the serum, one drop of the primary antibody (mouse anti-human S100 monoclonal antibody and 1:100 diluted c-kit) was applied and incubated for 1 h at 37°C. After rinsing with PBS three times, each for 5 min, the PBS was removed, and universal secondary antibody was added to each section and incubated at room temperature for 30 min. After rinsing with PBS three times, each for 3 min, the PBS was removed, and one drop of freshly prepared DAB solution was added to each section for observation under a microscope for 3–5 min. After rinsing with tap water, hematoxylin counterstaining was performed, followed by differentiation with 0.1% hydrochloric acid, rinsing with tap water, and PBS bluing. The sections were dehydrated through graded alcohol, clarified with xylene, and mounted with neutral resin for observation of Cajal-like cell counts under a microscope.

### 2.4 Data collection

Under a 400× optical microscope, 10 random fields in the muscle layer were selected, and imaging software (Tiny Eye labs, Copyright © 2017 Idée Inc., 223 Queen St. E., Toronto, ON, Canada M5A 1S2) was used to distinguish and calculate the specific content of muscle and collagen based on the differential staining (collagen appears blue, muscle appears red). The average value was taken, and the collagen-muscle ratio was calculated (12, 13). Cajal-like cells, which express the c-kit receptor tyrosine kinase on their surface, were identified through their positive reaction with the proto-oncogene c-kit antibody (14), allowing for an accurate count of Cajal-like cell counts. This study categorized the number of Cajal cells in each field into low density (0–1/HPF), medium density (2–3/HPF), and high density (≥4/HPF) (15), recording the number of fields in each density category.

### 2.5 Statistical methods

Quantitative data were analyzed using the *T*-test, whereas categorical data were assessed with the Chi-square test. A *P*-value of less than 0.05 was considered statistically significant.

## 3 Results

### 3.1 Clinical data

In this study, there were a total of 23 cases of intermittent hydronephrosis in children, including 19 boys and 4 girls, all of whom were confirmed to have unilateral ureteropelvic junction stricture during surgery. The median age at onset was 56 (range 0–96) months, the median age at the time of pyeloplasty was 81 (range 60–104) months, and the average time interval from detection to surgery was 28.4 ± 29.5 months. Among these cases, six patients were identified to have hydronephrosis during the fetal period, while the other 17 initially presented with abdominal pain. Twenty-one patients underwent left-sided dismembered pyeloplasty, and the remaining two had the procedure performed on the right side. Except for two patients, the rest had a history of varying degrees of flank pain (see Table 1). Before pyeloplasty, all children underwent ultrasonography, and it was observed that the anteroposterior diameter of the renal pelvis in the observation group was slightly larger than that in the control group, while the thickness of the renal cortex was slightly thinner than that in the observation group (51.3 ± 18.1 vs. 43.8 ± 11 mm, *P* = 0.04; 2.75 ± 1.2 vs. 3.3 ± 1.7 mm, *P* = 0.03).

**Table 1:** Clinical data of children with intermittent hydronephrosis.

	*n* (%)
Gender	
Boys	19 (82.6)
Girls	4 (17.4)
Affected side	
Left	21 (91.3)
Right	2 (8.7)
Flank pain	
No	2 (8.7)
Yes	21 (91.3)
Initial symptoms	
Prenatal examination	6 (26)
Consultation for flank pain	17 (74)
Total	23 (100)

### 3.2 Pathological data

**Figure 1:**
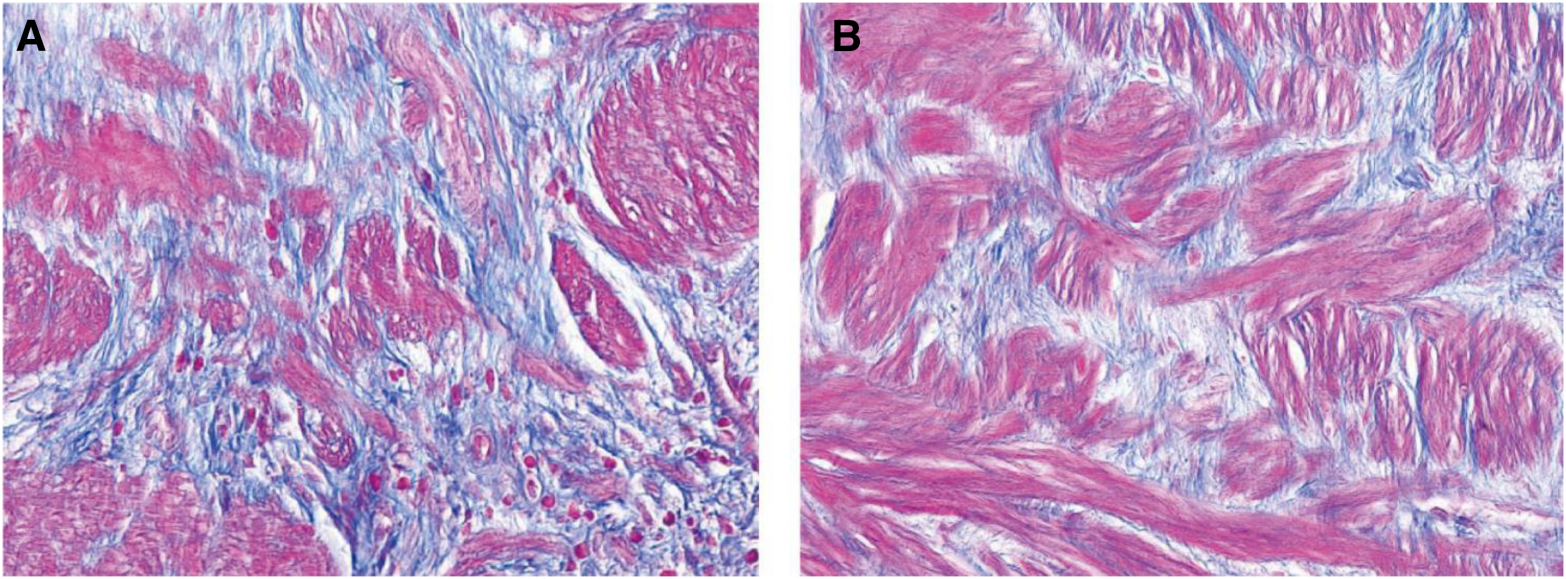
Differences between intermittent and persistent hydronephrosis specimens under masson's trichrome staining. (**A**) Shows a specimen with persistent hydronephrosis, where the muscle content is lower, the arrangement is disorganized, and there is a heavier deposition of collagen. (**B**) Represents a child with intermittent hydronephrosis, exhibiting comparatively higher muscle content, more orderly arrangement, and lighter collagen deposition.

This study summarized and compared the pathological data of 920 slides from 46 pediatric patients, including 460 Masson's trichrome stained slides and 46° c-kit immunohistochemical slides. Under Masson's trichrome staining, muscle tissue appears red, while the intervening collagen displays a blue hue. The collagen-to-muscle ratio (CMR) was determined by analyzing the area ratio of blue to red regions using an imaging software. Samples from children with intermittent hydronephrosis show a lower collagen content, higher muscle content, and a more regular alignment, whereas samples from children with persistent hydronephrosis contain a higher amount of collagen, lower muscle content and a more disordered arrangement (see Figure 1). The collagen-to-muscle ratio in children with intermittent hydronephrosis is significantly lower than those with persistent hydronephrosis (1.59 ± 0.65 vs. 3.98 ± 1.19, *P* < 0.001).

**Figure 2:**
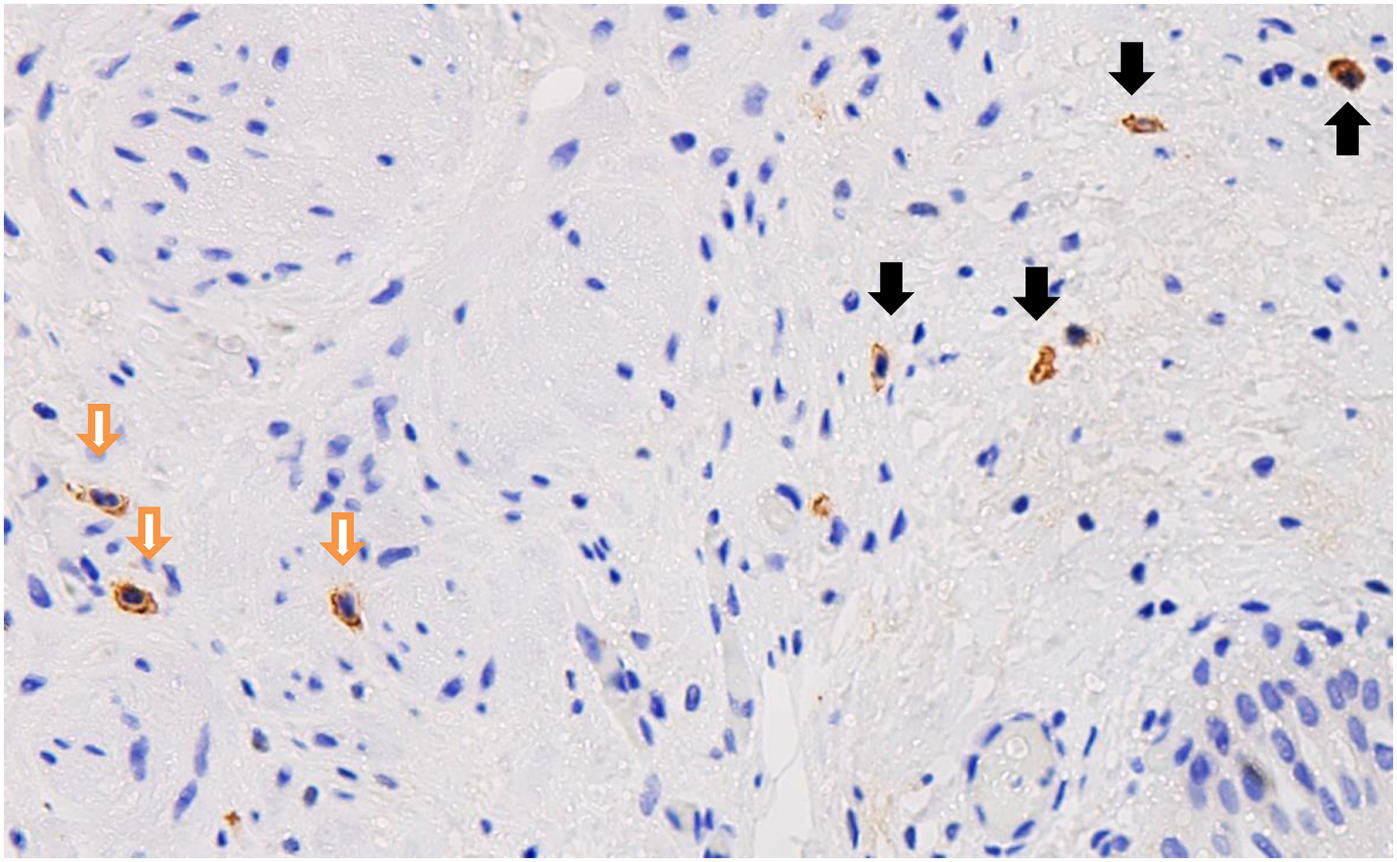
Following c-kit immunohistochemical staining, Cajal cells are brownish and spindle-shaped, distributed within the muscular layer (indicated by the white arrows); whereas, the brownish cells distributed in the submucosal layer are mast cells (indicated by the black arrows).

After c-kit immunohistochemical staining, Interstitial Cells of Cajal (ICCs) appeared brownish and spindle-shaped, located between the muscular layers. The confusingly similar mast cells also stained brownish, but presented mostly as round or oval shapes and distributed in the submucosal or serosal layers (see Figure 2). There was a significant difference between the number of low-density fields (0–1/HPF), medium-density fields (2–3/HPF), and high-density fields (≥4/HPF) of ICCs in samples from children with intermittent hydronephrosis compared to those with persistent hydronephrosis [132 (57.4%) vs. 173 (75.2%); 70 (30.4%) vs. 38 (16.5%); 28 (12.2%) vs. 19 (8.3%), *P* < 0.001]. The low-density ICC fields accounted for the majority in both groups; compared to persistent hydronephrosis, there were fewer low-density ICC fields in intermittent hydronephrosis [132 (57.4%) vs. 173 (75.2%)], more medium-density fields [70 (30.4%) Vs. 38 (16.5%)], but the number of high-density fields was similar [28 (12.2%) vs. 19 (8.3%)] (see Table 2).

**Table 2:** Number of fields with different density of Cajal-like cells *n* (%).

	Low-density	Medium-density	High-density
Persistent hydronephrosis	173 (75.2%)	38 (16.5%)	19 (8.3%)
Intermittent hydronephrosis	132 (57.4%)	70 (30.4%)	28 (12.2%)
*P*-value	<0.001		

## 4 Discussion

Intermittent hydronephrosis secondary to ureteropelvic junction obstruction (UPJO) is characterized by acute abdominal or flank pain during periods of hydronephrotic episodes, with or without accompanying nausea and vomiting, known as Dietl's crisis (7). During a Dietl's crisis, the anteroposterior diameter (APD) of the renal pelvis typically enlarges considerably, reducing automatically upon alleviation of pain. Imaging examinations of the urinary system show no abnormalities during intervals (5, 6). Hence, the diagnosis of intermittent hydronephrosis necessitates routine follow-up and early renal ultrasound assessment during and after a Dietl's crisis, with observation of the extent of hydronephrosis post-induction via exercise, excessive hydration, or diuretics if needed (16). Some studies suggest that in school-aged children, abdominal pain in approximately one per hundred individuals is caused by UPJO. A comprehensive medical history is key to differentiating this condition; any preschool-aged child with a history of recurrent abdominal pain should be suspected of having intermittent hydronephrosis (9). The principal causes of intermittent hydronephrosis are extrinsic compression by crossing vessels at the lower pole of the kidney (7) and ureteral polyps (8), while intrinsic reasons include congenital stricture and abnormal peristalsis at the ureteropelvic junction (9–11).

The pathogenesis of ureteropelvic junction obstruction (UPJO) is currently unclear, even the term “ureteropelvic junction” itself is merely a conceptual designation by urologists, not a fixed anatomical location (17). Possible etiologies involve structural and functional abnormalities. Structural anomalies pertain to obstructive lesions inside or outside the ureter, potentially including polyps, crossing vessels, stones, or tumors. Functional abnormalities typically correlate with intrinsic stricture and poor peristalsis at the ureteropelvic junction, possibly resulting from disordered muscle structure, atrophy of smooth muscle cells, increased collagen deposition in muscle layers, and reduction in the number of Interstitial Cells of Cajal (ICCs) (18–20). Muscular atrophy and collagen deposition (increased collagen to muscle ratio) are classic pathological changes in UPJO, and in this study, the collagen to muscle ratio in intermittent hydronephrosis was lower compared to persistent hydronephrosis, suggesting a milder degree of lesion at the ureteropelvic junction with greater compliance in children with intermittent hydronephrosis. Conversely, since children with intermittent hydronephrosis only manifest hydronephrosis during brief episodes and are indistinguishable from normal children during remission, their degree of collagen deposition and muscle atrophy is relatively minor.

In recent years, the theory that loss of the unidirectional peristaltic mechanism in the muscular layer of the ureteropelvic junction (UPJ) leading to the inability of urine to flow from the renal pelvis into the ureter has become increasingly accepted (21), though the mechanism of this unidirectional peristalsis remains unclear, Interstitial Cells of Cajal (ICCs) play a vital pacemaker role in promoting peristaltic waves and facilitating the downward flow of urine into the ureter (22). Initially discovered in the gastrointestinal smooth muscles, the reduction in the density of ICCs is associated with disorders of gastrointestinal motility, such as congenital pyloric stenosis, achalasia, and Hirschsprung's disease (23), and those located outside the gastrointestinal tract such as urinary tract and genital tract are referred to as Cajal-like cells (24). Cajal-like cells in the urinary tract, similar to their function in the intestines, play a significant role in both physiological and pathological urinary processes. They are possibly involved in pacing and ensuring the patency of the urinary tract through regulation of neurogenic transmission and electrical impulse propagation (14).

Many studies have discovered a decreased density of Cajal-like cells at the UPJ in hydronephrosis compared to the normal population, leading to the speculation that a reduction in ICC density and morphological changes can affect the renal pelvis and ureteral rhythmicity, reducing peristalsis and thus becoming a significant factor in the formation of UPJO (1, 25, 26). While the relationship between ICC numbers and UPJO remains controversial, most studies agree that a reduction in ICCs closely relates to changes in ureteral motility (25, 26). Babu et al. compared samples from children undergoing pyeloplasty to those from normal children and found a significantly lower number of Cajal-like cells in the UPJO specimens (12). Solari V discovered a substantial number of c-Kit positive ICCs within the musculature of a normal UPJ, whereas a marked reduction was noticeable in the UPJO specimens (27). Additionally, some studies suggest the pathogenesis of UPJO might be unrelated to ICC quantity but possibly linked to ICC function (1, 28). Koleda et al. even found an increased density of ICCs in UPJO samples, which could reflect a compensatory mechanism to boost ureteral peristalsis (15). The density of ICCs correlates with age, generally presumed to increase with age. However, in children with UPJO, ICC density is reduced and consistently lesser than that of the healthy population (29). Despite clinical differences between intermittent and persistent hydronephrosis, both result from stenosis at the UPJ, with low-density ICC fields still prevalent at 400× magnification, but significantly fewer than in persistent hydronephrosis cases {132 (57.4%) vs.173 (75.2%)}. Moreover, the number of medium-density ICC fields is higher in intermittent cases, while the high-density ICC field numbers show little difference.

Dismembered pyeloplasty stands as the standard procedure for treating UPJO, with a failure rate of about 5%–7% (30). Literature indicates the collagen-muscle ratio (CMR) and ICC density have prognostic significance on surgical outcomes; Kim et al. and Babu et al. found that a lower collagen content and higher muscle content (i.e., lower CMR) in UPJ hydronephrosis correlate with better surgical outcomes (12, 13). However, some scholars believe the pathological structure at UPJ is unrelated to the success rate of pyeloplasty, with surgical technique being the primary determinant (31, 32). Long-term, large-scale prospective studies are necessary for an accurate assessment of the impact of CMR and ICC density on surgical outcomes.

In summary, compared to persistent hydronephrosis, children with intermittent hydronephrosis exhibit a higher density of Cajal-like cells at the stenotic segment of the UPJ; less collagen content, and higher muscle content (lower collagen-muscle ratio).

## Data Availability

The datasets presented in this article are not readily available because the dataset is subject to the following restrictions: It can only be utilized for the purposes of this research and cannot be shared or used for any other projects without prior approval from the Capital Institute of Pediatrics Ethics Committee. Additionally, all personal identifiers have been removed to maintain the confidentiality of the subjects. Requests to access the datasets should be directed to Ma Yan, 627492610@qq.com.
